# Rethinking the influence of hydroelectric development on gene flow in a long-lived fish, the Lake Sturgeon *Acipenser fulvescens*

**DOI:** 10.1371/journal.pone.0174269

**Published:** 2017-03-22

**Authors:** Craig A. McDougall, Amy B. Welsh, Thierry Gosselin, W. Gary Anderson, Patrick A. Nelson

**Affiliations:** 1 North/South Consultants Inc., Winnipeg, Manitoba, Canada; 2 Department of Biological Sciences, University of Manitoba, Winnipeg, Canada; 3 Division of Forestry and Natural Resources, West Virginia University, Morgantown, West Virginia, United States of America; 4 Independent researcher, Otterburn Park, Québec, Canada; Pacific Northwest National Laboratory, UNITED STATES

## Abstract

Many hydroelectric dams have been in place for 50 - >100 years, which for most fish species means that enough generations have passed for fragmentation induced divergence to have accumulated. However, for long-lived species such as Lake Sturgeon, *Acipenser fulvescens*, it should be possible to discriminate between historical population structuring and contemporary gene flow and improve the broader understanding of anthropogenic influence. On the Winnipeg River, Manitoba, two hypotheses were tested: 1) Measureable quantities of former reservoir dwelling Lake Sturgeon now reside downstream of the Slave Falls Generating Station, and 2) genetically differentiated populations of Lake Sturgeon occur upstream and downstream, a result of historical structuring. Genetic methods based on ten microsatellite markers were employed, and simulations were conducted to provide context. With regards to contemporary upstream to downstream contributions, the inclusion of length-at-age data proved informative. Both pairwise relatedness and Bayesian clustering analysis substantiated that fast-growing outliers, apparently entrained after residing in the upstream reservoir for several years, accounted for ~15% of the Lake Sturgeon 525–750 mm fork length captured downstream. With regards to historical structuring, upstream and downstream populations were found to be differentiated (F_ST_ = 0.011, and 0.013–0.014 when fast-growing outliers were excluded), and heterozygosity metrics were higher for downstream versus upstream juveniles. Historical asymmetric (downstream) gene flow in the vicinity of the generating station was the most logical explanation for the observed genetic structuring. In this section of the Winnipeg River, construction of a major dam does not appear to have fragmented a previously panmictic Lake Sturgeon population, but alterations to habitat may be influencing upstream to downstream contributions in unexpected ways.

## Introduction

Deforestation, urbanization, hydroelectric dams, weirs, water diversions, and linear transport infrastructure have altered terrestrial and aquatic environments, leading to widespread habitat fragmentation and threatening biological diversity [1–5]. However, the consequences of these changes are not always obvious or consistent with expectations after the confounding influence of habitat loss is removed [6,7]. At the species level, population fragmentation results from the introduction of one or more barriers to volitional movement and/or effective dispersal, isolating portions of a group of individuals that previously interacted genetically; over time, lack of gene flow among isolated groups could result in increased rates of genetic drift, decreased genetic variability, inbreeding depression, and ultimately risk of population extinction [8–11].

Population fragmentation often results from habitat fragmentation, but the concepts are not equivocal. Contemporary habitat identified as fragmented may not preclude or restrict gene flow of a given species [7]. Conversely, the assumption that populations were historically panmictic (presumably via contiguous habitat) prior to anthropogenic habitat alterations can be problematic, as evidence for limited historical gene flow (which gives rise to population structure) has been observed in a variety of species [12–15], and can facilitate adaptation [16,17]. Thus, management initiatives designed to replicate historical connectivity may in some cases be based on the erroneous assumption of symmetric gene flow among groups.

In the absence of baseline data, researchers attempting to assess barrier-related impacts in habitats altered by human activities have generally relied on genetic methods, modelling, or reference studies [7,18–21]. Commonly used population genetic metrics (i.e., those similar to Wright's F_ST_) quantify differences in allele frequencies and heterozygosity among groups of individuals; the genetic signature of each individual reflects its entire lineage, and as such, these equilibrium metrics effectively synthesize generations of potentially non-random mating schemes, migration, and the impacts of chance events such as genetic mutations and demographic bottlenecks on patterns of between- and within-group variation [22–27]. Based on snapshot sampling, timelines are generally not obvious from these indirect measures of genetic connectivity, and therefore separation of historical and contemporary influences, such as anthropogenic habitat fragmentation, remains problematic [7,12,14,15,20,28–30].

The rationale is particularly relevant to riverine systems. Dams and weirs have altered rivers around the world, including many of the largest and most biologically diverse [5,31,32]. Large-scale hydroelectric development was underway c. 1900 [5,33] when stock/population structure in fishes was an emerging concept, and there was little consideration of the biological consequences on aquatic systems [34–36]. The "unit stock" has since evolved into a core tenet of fisheries management [37,38], and in accordance significant emphasis has been placed on identification and delineation methods [36,39,40]. With the proliferation of molecular methods and the identification of neutral genetic markers over the last few decades, population/stock structure and gene flow patterns in fish can be resolved with relative ease [41–44]. It is now apparent that riverine fish populations can exhibit structuring wholly or in part attributable to natural processes [45–48]. As such, quantification of hydroelectric/fragmentation impacts is complicated, because many dams have been in place for 50 - >100 years [5]. For most fish taxa, this is sufficient time (generations) for barrier induced divergence to have occurred [7]; however, this is likely not enough time for long-lived species like sturgeon (Acipenseridae spp.), and therefore, genetic structure observed today should primarily reflect historical processes and natural impediments to gene flow.

The Lake Sturgeon, *Acipenser fulvescens*, exhibits several life history characteristics that make it an ideal candidate for addressing questions about historical population structure and gene flow patterns in large riverine systems. Maturation of females does not occur until ~18–27 years of age, and life-spans of 50–80 years are common [49–53], with individuals as old as 154 years having been reported [54]. Spawning intervals for females and males ranging from 2–7 and 1–3 years, respectively, have typically been reported [49,50,55–57]. Generation time for Lake Sturgeon is considered to be in the range of 26–50 years [58], so even in the presence of dams constructed 100 years ago, insufficient generations have theoretically passed for genetic divergence driven by anthropogenic habitat fragmentation to be apparent using equilibrium metrics [7,59–62].

Unlike equilibrium metrics, which are influenced by heterozygosity and therefore the proportions of common alleles [63,64], pairwise relatedness estimators (and closely related parentage/sibship assignment) can be used to assess family-based substructure (e.g., parent-sibling, full-sibling and half-sibling identical-by-descent relationships) within populations by exploiting the information provided by polymorphic loci and rare alleles [63,65–70]. In Lake Sturgeon populations, equilibrium metrics should primarily capture the influence of historical processes [61,71], but family-based methods should be useful to address contemporary patterns [24,72], whether or not populations were historically structured. However, there are functional hurdles. First, Lake Sturgeon populations exhibit relatively low levels of genetic differentiation across the species range [71,73–75]. Second, the 12–14 available disomically inherited microsatellite loci have only moderate variability (average number of alleles = 6.58; [76]), meaning that the overall resolution provided by the current genetic toolkit is limited in the absence of supplementary population data. Third, Lake Sturgeon populations across North America are demographically depressed, primarily due to systematic overharvest [49,50,53,77,78]. Fortunately, as a function of longevity and overlapping generations, there is minimal evidence of reduced genetic diversity in diminished populations [61,71,74,75], but small numbers of fish may impede collection of sufficient samples needed to resolve historical and/or contemporary patterns within rivers.

A few rivers inhabited by long-lived sturgeons have been examined in the context of population structure. Welsh and McLeod [79] studied genetic patterns using microsatellite loci in the 30 km long Namakan River, and a lack of genetic differentiation among sample groups in combination with contemporary movement data indicated the presence of a single population residing within this undeveloped system. On the 1,271 km long Ottawa River, it is believed that a formerly panmictic Lake Sturgeon population is now subdivided into distinct segments bounded by dams, with little or no gene flow occurring among them [61]. Due to lack of movement of later life stages past Ottawa River hydroelectric generating stations, Lake Sturgeon gene flow might now only occur in the downstream direction via larval drift, if it occurs at all [61]. Conversely, genetic data of the similarly long-lived White Sturgeon *Acipenser transmontanus* in the Upper Columbia River indicated spatial population structure [48], likely a result of isolation by distance within the Columbia-Snake river complex as a whole [62]. On the undammed Fraser River, multiple White Sturgeon populations exist along the flow axis [62,80]. All of the aforementioned sturgeon studies have linked contemporary population structure to historical processes rather than recent fragmentation by dams. Considering the observed differences in population structure among rivers inhabited by these two sturgeon species, it appears that patterns of both historical and contemporary gene flow might differ by river system, potentially reflecting the configurations of individual dams and reservoirs, as well as the severity of falls and rapids that existed prior to development.

**Fig 1:**
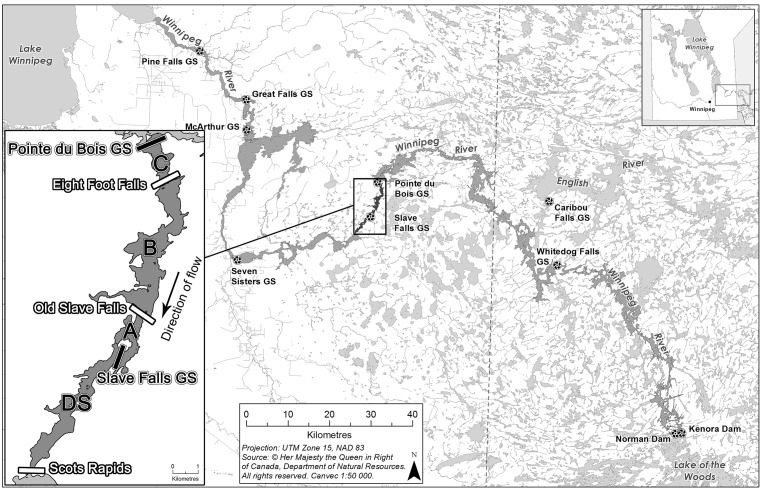
The Winnipeg River, from Lake of the Woods to Lake Winnipeg with hydroelectric generating stations marked. The Slave Falls study area is inset, with sampling zones (C, B, A, DS) and known movement restrictors labelled in white. The names "Eight Foot Falls and Old Slave Falls" reflect pre-impoundment conditions. Basemap source data provided by Natural Resources Canada, reproduced in accordance with an Open Government Licence—Canada (http://open.canada.ca/en/open-government-licence-canada).

On the Manitoba portion of the 260 km long Winnipeg River (Fig 1), Lake Sturgeon recruitment persists in all the hydroelectric reservoirs [81–83], but concerns over assumed fragmentation and reduced gene flow remain unaddressed [84,85]. Dams (7–18.6 m head differentials) currently prevent upstream movement of resident fish species among Winnipeg River reservoirs; however, these dams were constructed at or near the sites of historical falls and rapids, which, interspersed with riverine and lacustrine sections, were common from Lake of the Woods downstream to Lake Winnipeg [86,87]. While historical downstream redistribution of Lake Sturgeon seems likely, the falls/rapids may also have been natural barriers to upstream fish movement [88], resulting in a pattern of asymmetric gene flow pre-dating hydroelectric development. Juvenile, subadult and adult Lake Sturgeon from the Winnipeg River exhibit restricted movement patterns, influenced or even defined by in-stream habitat features such as natural or inundated falls/rapids [89–91], but at the Slave Falls Generating Station (GS), survived entrainment of subadult and adult Lake Sturgeon is common [92], suggesting a contemporary downstream gene flow scenario markedly different from that observed on the Ottawa River [61,93].

To improve the understanding of contemporary and historical Lake Sturgeon gene flow on the Winnipeg River, two hypotheses were tested:

1. Measureable quantities of former Slave Falls Reservoir Lake Sturgeon reside downstream of the Slave Falls GS. Support for this hypothesis would indicate probable contemporary gene flow across the putative barrier.
2. Genetically differentiated populations of Lake Sturgeon occur upstream and downstream of the Slave Falls GS. Support for this hypothesis would indicate that there was minimal (if any) upstream gene flow prior to dam construction.

A combination of equilibrium metrics and family-based genetic methods were used in conjunction with biological data, focusing on growth rate variation, from Lake Sturgeon upstream and downstream of the stations to address these hypotheses. To provide context for empirical results, population genetic simulations that accounted for the life history strategy of the species were conducted.

## Materials and methods

### Study area

The Winnipeg River flows from Lake of the Woods, Ontario to Lake Winnipeg, Manitoba [94,95]. Historically comprised of a series of lakes separated by short, high-gradient stretches, the Winnipeg River drops 105 m over its 260 km length [86]. The river's numerous falls/rapids (up to 15 m high) made the Winnipeg River desirable for hydroelectric development during the first half of the 20^th^ century [33,86]. The Winnipeg River now consists of a series of cascading reservoirs, defined by the nine generating stations that produce power. Six lie in the province of Manitoba, while the remaining three are upstream in Ontario (Fig 1). Near the end of its course, Winnipeg River discharge averages 993 m^3^s^-1^ (range: 135 to 2990) [96].

This study was conducted in the Slave Falls Reservoir and the section of river located downstream (Fig 1). The Pointe du Bois GS (established in 1909; 50°17’52N, 95°32’51W) is located at the upstream end. The Slave Falls GS (established in 1931; 50°13’25N, 95°34’02W) backwaters the downstream end of the reservoir and, due to its 9.5 m head/waterfall drop and associated infrastructure, is a contemporary barrier to upstream fish passage. However, prior to dam construction, the river plunged ~6 m at Old Slave Falls [86].

As of 2007, the Slave Falls Reservoir was estimated to support ~2,205 adult Lake Sturgeon (95% CI: 921–4,095) based on mark-recapture methods (Manitoba Hydro unpublished data). For consistency with previous research, the Slave Falls Reservoir was subdivided into sampling zones that account for movement patterns of Lake Sturgeon being restricted by the inundated falls/rapids present in the reach [90–92]. Adult sampling was conducted below the only known spawning location in the reservoir, at the upstream end of Zone C (1.8 river kilometers [rkm]; Pointe du Bois downstream to Eight Foot Falls; Fig 1). Juvenile/subadult sampling within the Slave Falls Reservoir was conducted further downstream in zones B (6 rkm; Eight Foot Falls to Old Slave Falls, whose names reflect pre-impoundment conditions) and A (2.1 rkm; Old Slave Falls to the Slave Falls GS). The final sampling zone was a 6 km reach stretching from the Slave Falls GS downstream to Scot Rapids, denoted Zone DS (Fig 1). A population estimate has never been conducted in this area, but it is clear that high densities of juvenile Lake Sturgeon inhabit the area [81,97], and up to 270 adults, aggregated for spawning, have been captured in the area during a single spring using large-mesh gillnets (Manitoba Hydro unpublished data); many more undoubtedly eluded capture. Extensive Lake Sturgeon egg deposition has also been observed downstream of both the Slave Falls GS powerhouse and spillway (Manitoba Hydro unpublished data). Scots Rapids restricts upstream and downstream movement of juveniles [89]. The next generating station (Seven Sisters GS) is located ~41 km downstream of the Slave Falls GS (Fig 1).

### Fish collection

Adult Lake Sturgeon (>800 mm fork length [FL]; [90]) were captured using large-mesh gillnets of various lengths (22.9–91.6 m) between May 25 and June 9, 2009. Stretched meshes measuring 203, 229, 254, and 309 mm (equal effort by mesh size) were used. Each Lake Sturgeon captured was measured for FL, total length, and body mass. Each untagged adult received a unique Floy^®^ tag, inserted between the basal pterygiophores. Numbers of previously tagged fish were also recorded. External sex identification methods were consistent with those used by Dumont et al. [98]; males were identified by the presence of milt, while females were identified based on the presence of eggs, and/or a cloaca that was swollen, red and distended. Adult sized fish captured at the spawning site but lacking the aforementioned characteristics were considered "unknown". For each fish from which sex could be determined, a 2-cm^2^ fin clip was removed from the lower lobe of the caudal fin, and placed into an individually labelled vial filled with biological grade (95%) ethyl alcohol for subsequent genetic analysis.

To maximize the potential for relatedness (i.e., family relationships), several analyses were designed to focus on juvenile/subadult Lake Sturgeon within a narrow age range. Upstream of the Slave Falls GS (zones A and B; Fig 1), fish between 550 and 750 mm FL were targeted. Gill nets (91.6 m long x 1.8 m deep, 127 mm stretched nylon mesh) were set overnight between May 19 and July 21, 2010, in deep water habitats (15–40 m) known to be utilized by this size-class in the Slave Falls Reservoir [83,90,92]. Biological measurements and tissue samples were collected as per adults. Approximately 50% of the upstream juveniles sampled were marked with an individually numbered Floy^®^ tag. The first ray of the left pectoral fin, immediately distal to the fin articulation, was removed for ageing and placed in an individually labelled envelope, prior to the fish being released. Upon returning from the field, genetic samples were stored in a -20°C freezer and fin rays were dried at room temperature.

Downstream of the Slave Falls GS (Zone DS; Fig 1), Lake Sturgeon between 450 and 750 mm FL were targeted, since juvenile growth rate was known to be slower in this section of river [97]. Gill nets were set overnight between August 4 and 20, 2010, in areas where water depths were >13.9 m, as suggested by Barth et al. [81]. Sampling methods were consistent with those used in upstream zones A and B, except that a variety of mesh sizes (51–152 mm) and net lengths were employed, Floy^®^ tags were not applied, and a fin clip was removed from the right pelvic fin (as opposed to the caudal fin) so that upstream and downstream sampled fish could be distinguished. Recaptured fish identified as having originated from upstream of Slave Falls based on Floy^®^ tags or fin clips were noted and included in downstream sampling. Although larger individuals from the Slave Falls Reservoir would more aptly be classified as subadults [83,90], all Lake Sturgeon <800 mm FL sampled in the current study are hereafter referred to as juveniles for ease of notation.

Lake Sturgeon fin rays were air dried, mounted in epoxy, and sectioned using a Struers Minitom low-speed saw (Struers Inc, Cleveland, Ohio). Two 0.6 mm sections were cut, placed on an individually labelled glass slide and coated in Cytoseal^™^ 60 (Thermo Scientific, Waltham, Massachusetts). Under 30 – 40x magnification, fin ray sections were aged (annuli counted) without knowledge of the length, weight, or tag numbers of the individual fish. Each structure was read twice by the lead author and once by an experienced technician. Final ages were determined by modal consensus of the three readings. Only downstream fish <12 years old were assigned ages, as those older tended to lack the annuli separation needed for confident age determination.

In order to evaluate the discriminatory potential of family-based methods based on the level of genetic resolution afforded by the available suite of microsatellite markers for Lake Sturgeon, samples were also collected from known full-sibling, half-sibling and unrelated Lake Sturgeon, generated from wild Slave Falls Reservoir parents and raised in a University of Manitoba hatchery (C. Klassen, University of Manitoba, unpublished data). In spring 2009, larvae were generated by fertilizing the eggs from each of two females with the milt of a different male, in order to generate two full-sibling families (unrelated at the first generation). In fall 2010, fin clips were collected from 15 fish from each of the two families, and preserved in ethyl alcohol. In spring 2010, additional Lake Sturgeon larvae were generated by fertilizing the eggs of two wild females with the milt of five wild males, all from the Slave Falls Reservoir. Two larvae from each of the 10 parental combinations (i.e., full-sibling and half-sibling relationships were generated) were preserved in 95% ethyl alcohol for subsequent genetic analysis. Genetic samples were also collected from 2010 broodstock parents (two females, five males), as well as three additional males whose gametes were not ultimately used.

Lake Sturgeon capture and sampling was authorized under Manitoba Water Stewardship Scientific Collection Permits #04–09 and #15–10. All described methods were conducted under animal care protocols approved by the Protocol Management Review Committee at the University of Manitoba, based on the guidelines of the Canadian Council for Animal Care.

### DNA extraction

DNA was extracted from samples using the Promega Wizard SV 96 Genomic DNA Purification System (Promega Corporation, Madison, Wisconsin) according to the manufacturer’s protocol. Fin-clip samples were eluted in 500 μl of nuclease-free water and larval samples were eluted in 50 μl of water. Twelve microsatellite loci (*AfuG 9*, *AfuG 56*, *AfuG 63*, *AfuG 74*, *AfuG 112*, *AfuG 160*, *AfuG 195*, *AfuG 204*, *Afu 68*, *Afu 68b*, *Spl 120*, *Aox 27*; described in Welsh and May [76]) were amplified in the first batch of 50 samples. *Aox 27* and *AfuG 204* were monomorphic in all 50 samples, as observed previously in other Hudson Bay drainage basin localities [75,79], so these loci were not analyzed in the remaining samples. Polymerase chain reaction (PCR) conditions are described in Welsh and McLeod [79]. PCR products were pooled into four groups and visualized on a Beckman Coulter CEQ 8000 Genetic Analysis System (Beckman Coulter Incorporated, Brea, California). Alleles were scored according to standardized designations [76]. A maximum of two alleles were observed per loci for each fish sampled.

### Data analysis

#### Biological data

Length-at-age, and gillnet catch-per-unit-effort (CPUE) were compared among the three juvenile sampling zones, from upstream to downstream: Zone B, Zone A, and Zone DS. Trends in length-at-age were assessed by age class. Statistical analysis of biological data was conducted using Analysis of Variance (ANOVA) and Tukey’s honestly significant difference (HSD) test. Gillnet CPUE values were compared between upstream Zones A and B using a Wilcoxon Rank Sum Test, due to non-normality. Zone DS was omitted from the CPUE comparison due to mesh-size inconsistencies.

#### Population genetics

Equilibrium genetic metrics such as F_ST_, heterozygosity, and allelic richness, which have been used to assess connectivity and population structure in sturgeon populations from natural and hydroelectrically developed rivers [62,79], were used to quantify population structure upstream (adults, juveniles) and downstream (juveniles) of the Slave Falls GS. Observed heterozygosity (H_O_), expected heterozygosity (H_E_), and F_ST_ were calculated in Arlequin version 3.5 [99]. F_ST_ was assessed for significance based on 10,000 permutations. Loci were tested for Hardy-Weinberg equilibrium in Arlequin. Locus pairs were tested for linkage disequilibrium in GENEPOP version 4.1 [100,101] using settings of 10,000 dememorization steps, with 200 batches and 10,000 permutations per batch. Standardized allelic richness (A_S_), private allelic richness (P_As_) and total allele counts were calculated using HP-Rare [102]. GENEPOP was also used to calculate inbreeding coefficient (F_IS_). Differences in observed heterozygosity of juvenile groups (i.e., upstream and downstream) were assessed based on proportions of loci found to be heterozygous for each individual genotyped [103]; given observed data normality, significance was tested using ANOVA. Differences in H_E_ and allelic richness between juvenile size classes were assessed using one-tailed Student *t* tests. Significance of multiple comparisons (Hardy-Weinberg equilibrium, linkage disequilibrium and F_ST_) was addressed using sequential Bonferroni corrections [104]. Effective population size (N_e_) was calculated by group (upstream adults, upstream juveniles, downstream juveniles) using the linkage disequilibrium method (lowest allele frequency used = 0.001 and parametric 95% confidence intervals) in NeEstimator v2 [105]. It should be noted that N_e_ estimates based on juveniles tend to be biased toward N_b_ (the effective number of breeders in one cycle of reproduction) should progeny from only a fraction of the previous generation parents be sampled [106]; however, Duong et al. [107] found that the harmonic mean of single cohort N_e_ estimates, based on the linkage disequilibrium method, for Lake Sturgeon approximated the N_e_ estimate based on all ~800 adults sampled from the population over a 10 year period. As such, bias in N_e_ estimates based on juvenile Lake Sturgeon samples is expected to be minimal, especially if multiple cohorts are combined.

#### Pairwise relatedness

Pairwise relatedness estimators have been utilized in a variety of applications, including quantifying inbreeding and dispersal [108], assessing kin association [72], and revealing disparate contributions of parents to cohort strength [24,109]. Herein, pairwise relatedness estimators in combination with biological data were used to ascertain downstream captured juveniles suspected to have been entrained at Slave Falls following years of upstream residence. Based on control samples of known pedigree (full-sibling, half-sibling, and unrelated at the first generation) and allelic frequencies of Slave Falls Reservoir adults, pairwise relatedness distributions were generated using seven common relatedness estimators implemented in COANCESTRY [110]. The following estimators were used: 1) TrioML, a triadic maximum likelihood estimator [111]; 2) Wang’s estimator, a moment estimator [112]; 3) Lynch’s estimator, a moment estimator [68]; 4) Lynch and Ritland’s estimator, a moment estimator [69]; 5) Ritland’s estimator, a moment estimator [66]; 6) Queller and Goodnight’s estimator, a moment estimator [67]; and 7) Milligan’s estimator, a dyadic maximum likelihood estimator [113]. Final COANCESTRY runs were conducted using 1000 reference individuals and 1000 bootstrapping samples. In total, 220 full-sibling, 100 half-sibling and 905 unrelated pairwise combinations were generated for each of the seven estimators. The relative ability of the estimators to resolve differences in *a priori* relatedness was assessed using the χ^2^ value associated with Kruksal-Wallis non-parametric tests and the maximum probability value associated with Steel-Dwass All Pairs test of multiple comparisons (i.e., full-sib vs. half sib, full-sib vs. unrelated, half-sib vs. unrelated). Strong estimators were then used to generate relatedness values for pairwise combinations of juvenile field samples in COANCESTRY. Parameters for the final field run were the same as those used to generate control distributions, except that allelic frequency was calculated from all juvenile samples analyzed (i.e., assumed panmixia). Statistical significance of differences in pairwise relatedness distributions among field groups was tested using Wilcoxon Rank Sum (2 groups) or Kruksal-Wallis (3 or more groups) tests, with multiple comparisons of the latter evaluated using Steel-Dwass All Pairs method.

Statistical analyses described thus far were conducted using JMP 8.0 (SAS Software, Cary, North Carolina), and a significance level (α) of 0.05 was used.

#### Bayesian inference

STRUCTURE version 2.3.4 [114,115] was used to assess the likelihood of population structuring patterns within the study area. Analyses were conducted using only juvenile genotype data, as well as with priors (sampling location and length-at-age). The admixture model and correlated allelic frequencies were used for all analyses. Putative populations (k) of 1–4 were tested, and 30 iterations were run per k. Burn-in period and re-sampling reps were both set to100,000. Default settings were used for all other parameters. Results were synthesized using STRUCTURE HARVESTER [116]. Significant differences between the most probable prior/k combinations were tested using the Steel-Dwass All Pairs method, due to a lack of homogeneity of variance.

#### Population simulations

Simulations were conducted using an individual-based, forward-time engine that allows complex life history and demography scenarios, RMETASIM package v.3.0.6 [117]. Simulations were based on the premise of an ancestral panmictic population, and subsequent separation into two populations (upstream and downstream). Barriers of differing permeability were incorporated by varying the quantity of inter-population dispersal (specifically, no-gene flow in either direction or asymmetric gene flow), scenarios potentially relevant to the interpretation of empirical study results.

To account for the life history strategy of the Lake Sturgeon (i.e., late maturation, iteroparity, longevity), individuals were placed into a 100 stage population matrix [118,119], with each stage corresponding to one year. Mortality in Lake Sturgeon is very high during the first year of life and low thereafter [52,58,120–123], so the 0–1 age/stage was bypassed to simplify computations. Lake Sturgeon were considered juveniles until age 24, and adults between age 25 and 100; mortality rates are presented in S1 Table. An equal sex ratio was assumed. Reproductive values for adults were selected to account for differing spawning periodicity of females and males [55], and scaled so as to achieve slightly positive growth. However, stochasticity in reproduction and survival was implemented based on a uniform distribution, resulting in variance around the expected number of individuals in each stage at any given time point within the simulation. At the end of each year, if the population size exceeded carrying capacity, population regulation occurred by randomly removing individuals, regardless of demographic stage, until the population size fell below the prescribed carrying capacity. The following five census (N_C_) population sizes (carrying capacities) were explored: 250, 500, 1,000, 2,000 and 3,000.

The final recession of Glacial Lake Agassiz occurred approximately ~7,500 years B.P [124–127], which would theoretically set the timeline for historical population divergence of Lake Sturgeon on the Winnipeg River in the absence of pre-hydroelectric development population panmixia. Simulations spanning a period of 8,750 years were constructed, the premise being that years 0 to 7500 would correspond to the past, year 7500 would be the present day, and years 7501 to 8750 would correspond to the near future. In Scenario 1, the two populations were isolated (no dispersal occurred between them) from year 0 to 8750. In Scenario 2, a quantity of upstream to downstream dispersal consistent with results observed empirically herein was applied from year 0 to 8750. In Scenario 3, upstream and downstream populations were isolated until year 7425 (i.e., designed to correspond with the approximate timeline of Slave Falls GS construction), after which the empirically observed quantity of upstream to downstream dispersal was applied. In the two scenarios that incorporated dispersal, the process was restricted to fish aged 5 to 12 years old, reflecting a conservative simplification of empirical observations. Scenario 3 was designed to assess if observed levels of upstream to downstream dispersal would be compatible with moderate levels of genetic differentiation persisting between populations.

Initial multilocus genotypes (i.e., individuals) were generated *in silico* based on 10 unlinked microsatellite loci, randomly drawn from the overall allelic frequency distribution observed empirically. Offspring genotypes were based on random mating, independently segregating alleles, and neutrality of markers. Because nothing is known about Lake Sturgeon microsatellite mutation rates (μ), simulations were conducted using a range of values (1e^-05^ to 1e^-02^) that included the commonly used default (1e^-04^). A single-step mutation model was used, without any restriction on repeat accumulation.

Every 25 years, genotype data from all individuals were harvested and F_ST_ [25] calculated. Each of the 60 different parameter combinations were replicated 32 times. F_ST_ results were plotted as means with 95% confidence intervals.

## Results

### Biological data

During spring 2009, large-mesh gill nets set at the Pointe du Bois GS spawning location yielded the capture of 345 (1007 ± 128 mm FL) individual Lake Sturgeon (454 total captures). Tissue samples were collected from 199 individuals identified as current year spawners.

A total of 535 juvenile Lake Sturgeon were captured during summer/fall gill netting: 145 from Zone B, 80 from Zone A and 310 from Zone DS. CPUE in Zone B (mean = 33.4 sturgeon/100 m/24 h, range: 21.0–51.4) was greater than in Zone A (mean = 3.0 sturgeon/100 m/24 h, range: 0–13.4) (Wilcoxon Rank Sum test, Z = 3.55, p = 0.0004).

Juvenile Lake Sturgeon captured from Zones A and B were between 7 and 10 years old, so as to maximize the potential for sibling relationships, only those from Zone DS that were 6–11 years old (n = 183) were included in subsequent genetic analysis. Length-at-age analysis indicated that fish from Zones A and B tended to be larger for a given age compared to Zone DS (Table 1). Significant differences in growth rate were found for age-7 (ANOVA, F_2,46_ = 33.7, p < 0.0001), age-8 (ANOVA, F_2,232_ = 282.0, p < 0.0001), age-9 (ANOVA, F_2,92_ = 182.4, p < 0.0001) and age-10 Lake Sturgeon (ANOVA, F_1,11_ = 31.8, p = 0.0002). Also, Zone A fish tended to be larger for a given age than Zone B fish, although differences were only significant for age-8 fish. No Lake Sturgeon ≤ 5 years old were identified in the downstream catch.

**Table 1:** Comparison of length-at-age for Lake Sturgeon captured in the three sampling zones, by age class. Data is summarized by number of fish (n), mean fork length (FL), standard deviation of fork length (SD), and ranked order of significant differences as determined by Tukey`s HSD (Rank). Zones which share the same rank (i.e., 1, 2 or 3) are not significantly different.

Zone	Age-7				Age-8				Age-9				Age-10			
	n	FL	SD	Rank	n	FL	SD	Rank	n	FL	SD	Rank	n	FL	SD	Rank
B	16	583	26.1	1	95	603	33.2	2	30	637	36.2	1	-	-	-	-
A	2	610	8.5	1	64	621	42.4	1	10	655	45.0	1	2	697	5.7	1
DS	31	490	45.0	3	76	483	42.0	3	55	486	40.0	3	11	507	45.9	2

**Fig 2:**
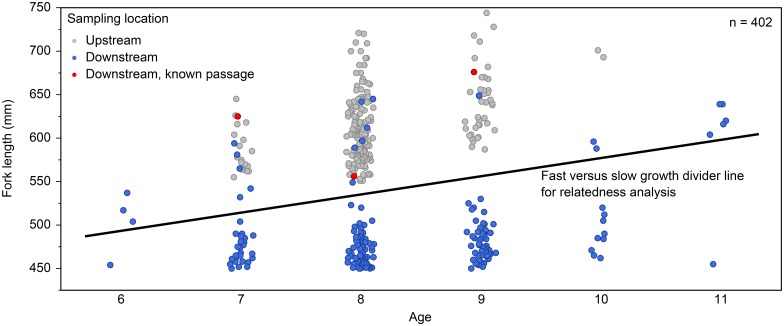
Length-at-age data for Lake Sturgeon ages 6–11, captured during the study, by location. Three fish known to have arrived downstream following entrainment at the Slave Falls GS based on mark-recapture are indicated.

Length-at-age analysis also revealed fast-growing outliers (n = 25) in the downstream population (Fig 2). During laboratory ageing, many of these fish were noted as having widely spaced annuli, similar to those observed in upstream fish and markedly different from the majority of the downstream fish. Three of these fast-growing outliers were identified as having descended over the Slave Falls GS between 2009 and 2010 based on mark-recapture (i.e., Floy^®^ tags or caudal fin-clips). Therefore, it was suspected that the other fast-growing outliers may also have been former Slave Falls Reservoir residents. Including fish ≥ 12 years of age, 23 of the 151 Lake Sturgeon between 525 and 750 mm FL (subadults) captured in Zone DS, or 15.2% (95% confidence interval of 10.4–21.8%), were fast-growing outliers.

### Genetic samples

In addition to the Lake Sturgeon collected for broodstock purposes in 2010 (n = 10), a random subsample of Slave Falls Reservoir adults (n = 120) identified as current year spawners in the spring 2009 gillnet catch were genotyped. All juveniles captured in Zone A (n = 80) were included, as were a random sub-sample (n = 51) of those captured in Zone B. Of the 183 juveniles from Zone DS that met the criterion of being 6–11 years old, all fast-growing outliers (n = 25) and a random sub-sample (n = 99) of the slow-growing juveniles were included. In total, microsatellite analysis was conducted on 130 adult, 131 upstream juvenile and 124 downstream juvenile samples. Samples missing data at three or more loci (n = 9) were excluded from subsequent analysis. The breakdown of the genetic dataset is summarized in Table 2.

**Table 2:** Summary of genetic data, with number of samples (n), mean number of alleles observed per locus (A_n_), allelic richness, standardized to 40 genes (A_s_), private allelic richness (P_As_), expected heterozygosity (H_E_), observed heterozygosity (H_O_), inbreeding coefficient (F_IS_) and effective population size (N_e_). Fast-growing and slow-growing downstream sub-groups assigned as per Fig 2.

Group	n	A_n_	A_s_	P_As_	H_E_	H_O_	F_IS_	N_e_
Upstream adults	129	4.9	4.16	0.23	0.595	0.606	-0.019	352 (166–6683)
Upstream juveniles	124	5.1	4.15	0.15	0.586	0.556	0.051	383 (171 - ∞)
Downstream juveniles	123	5.1	4.31	0.43	0.610	0.609	0.002	713 (226 - ∞)
Fast-growing	25	4.2	4.11	0.06	0.576	0.578	-0.005	-
Slow-growing	98	5.1	4.33	0.34	0.619	0.617	0.003	-

### Population genetics

The total number of alleles per polymorphic locus ranged from 2 (*AfuG195*) to 9 (*Afu68*, *AfuG9*, *Spl120*), for an average of 5.9 alleles per locus. F_ST_ did not vary significantly for juveniles captured in Zone A and Zone B (F_ST_ = 0.005, p = 0.064 ± 0.009), so these two upstream zones were pooled for subsequent analysis. Only *Spl120* (upstream adults) and *AfuG9* (upstream juveniles) deviated from Hardy-Weinberg equilibrium (p = 0.0004 and 0.0034, respectively) after sequential Bonferroni correction (α = 0.005). The only significant linkage disequilibrium occurred between *AfuG112* and *AfuG63* (p < 0.0001) in the upstream juvenile group (α = 0.001).

Upstream (i.e., zones A + B pooled) and downstream juveniles were found to be genetically differentiated (F_ST_ = 0.0108, p < 0.0001) when all downstream samples were included in the analysis (Table 3). Genetic differentiation was also found between upstream adults and downstream juveniles (F_ST_ = 0.0114, p < 0.0001). No significant differentiation was observed between upstream adults and upstream juveniles (F_ST_ = 0.00142, p = 0.133 ± 0.003). Observed heterozygosity of juveniles was significantly greater downstream (H_O_ = 0.609) than upstream (H_O_ = 0.556; ANOVA, F_1,245_ = 8.14, p = 0.0047). Expected heterozygosity of juveniles was significantly greater downstream (H_E_ = 0.610) than upstream (H_E_ = 0.586; *t*_*9*_ = 1.91, p = 0.044). Standardized allelic richness was not significantly different between downstream and upstream juveniles (*t*_*9*_ = 1.08, p = 0.15). Mean estimates of effective population size (N_e_) ranged from 352–713 for the three primary groups of fish analyzed (Table 2).

**Table 3:** Pairwise comparisons of F_ST_ between Lake Sturgeon sample groups. Fast-growing and slow-growing downstream sub-groups assigned as per Fig 2. Statistical significance was evaluated after a sequential Bonferonni correction (α = 0.008). Statistically significant comparisons are shown in bold.

Group 1	Group 2	F_ST_	p
Upstream adults	Upstream juveniles	0.00142	0.133 ± 0.003
**Upstream adults**	**Downstream juveniles**	**0.0114**	**0.0000 ± 0.0000**
Upstream adults	Fast-growing downstream	0.00661	0.0443 ± 0.002
**Upstream adults**	** Slow-growing downstream**	**0.0134**	**0.0000 ± 0.0000**
**Upstream juveniles**	**Downstream juveniles**	**0.0108**	**0.0000 ± 0.0000**
Upstream juveniles	Fast-growing downstream	0.00116	0.377 ± 0.005
**Upstream juveniles**	** Slow-growing downstream**	**0.0141**	**0.0000 ± 0.0000**
Fast-growing downstream	Slow-growing downstream	0.00353	0.152 ± 0.004

When downstream juveniles were separated by growth pattern (fast and slow), the fast-growing juveniles were not genetically differentiated from upstream adults (F_ST_ = 0.00661, p = 0.0443 ± 0.002) or upstream juveniles (F_ST_ = 0.00116, p = 0.377 ± 0.005) after sequential Bonferroni correction (α = 0.008) (Table 3). There was also no genetic differentiation between fast- and slow-growing downstream juveniles (F_ST_ = 0.00353, p = 0.152 ± 0.004). Conversely, the slow-growing downstream juveniles were differentiated from both the upstream adults (F_ST_ = 0.0134, p < 0.0001) and upstream juveniles (F_ST_ = 0.0141, p < 0.0001). Observed heterozygosity was significantly greater for slow-growing downstream (H_O_ = 0.617) versus upstream juveniles (H_O_ = 0.556; ANOVA, F_1,220_ = 9.36, p = 0.0025). Expected heterozygosity was significantly greater for slow-growing downstream (H_E_ = 0.619) versus upstream juveniles (H_E_ = 0.586; *t*_*9*_ = 2.52, p = 0.016). Standardized allelic richness was not significantly different between slow-growing downstream and upstream juveniles (*t*_*9*_ = 1.07, p = 0.15) (Table 2).

### Pairwise relatedness

**Fig 3:**
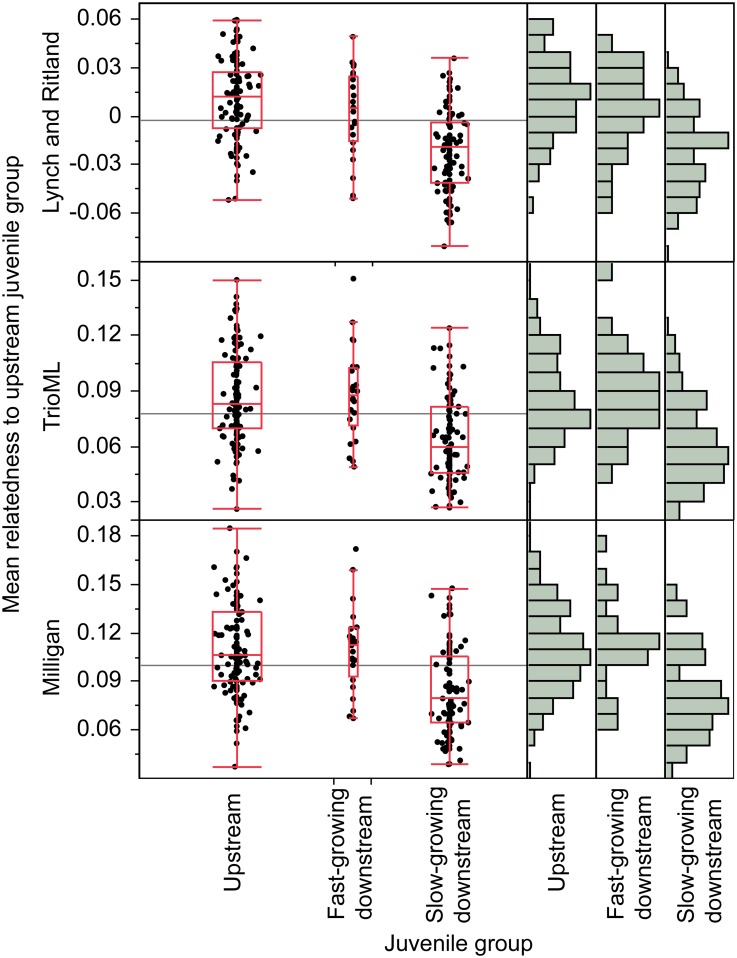
Mean relatedness of upstream (n = 124), fast-growing downstream (n = 25) and slow-growing downstream (n = 98) juvenile individuals with the upstream group. Kruksal-Wallis tests indicated significant differences (p<0.0001) for all three strong estimators (Lynch and Ritland, TrioML, Milligan). Steel Dwass All Pairs method consistently indicated that the slow-growing downstream juveniles were significantly less related to the upstream group than were the upstream and downstream fast-growing juveniles (S4 Table). Data are summarized using box plots and frequency distributions.

Pairwise relatedness distributions (full-sibling, half-sibling, or unrelated) of control fish were best described, in decreasing order, by the Lynch and Ritland [69], TrioML [111], and Milligan [113] estimators (S2 Table). Mean relatedness of upstream juveniles was higher than for downstream juveniles using the three strong estimators (S3 Table). Mean relatedness of each juvenile with all upstream juveniles was also calculated. Relatedness of upstream juveniles (n = 124), fast-growing downstream juveniles (n = 25), and slow-growing downstream juveniles (n = 98) with the upstream group were found to be significantly different, based on the Lynch and Ritland estimator (Kruksal-Wallis, p < 0.0001). A Steel-Dwass All Pairs test indicated that the slow-growing downstream juveniles tended to be less related to the upstream group than were either the fast-growing downstream juveniles or the upstream juveniles (Fig 3). Mean relatedness of upstream fish and fast-growing downstream fish to the upstream group were not significantly different. This pattern was consistent for the other two strong estimators (TrioML and Milligan; Kruksal-Wallis, p <0.0001; S4 Table).

### Bayesian inference

**Fig 4:**
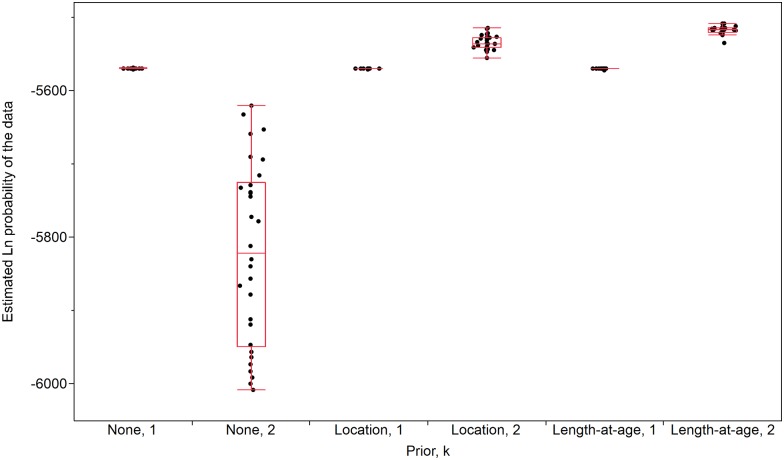
Results from STRUCTURE analysis used to identify the most likely Lake Sturgeon population structuring patterns in the vicinity of the Slave Falls GS, based on 10 microsatellite loci. Genotype data alone (none), and priors (sampling location [location], length-at-age) were considered in combination with putative populations (*k*) of 1–4. For illustrative purposes, only the most statistically probable scenarios (k = 1, 2) are shown. The synthesis of 30 iterations per combination is summarized using box plots.

**Fig 5:**
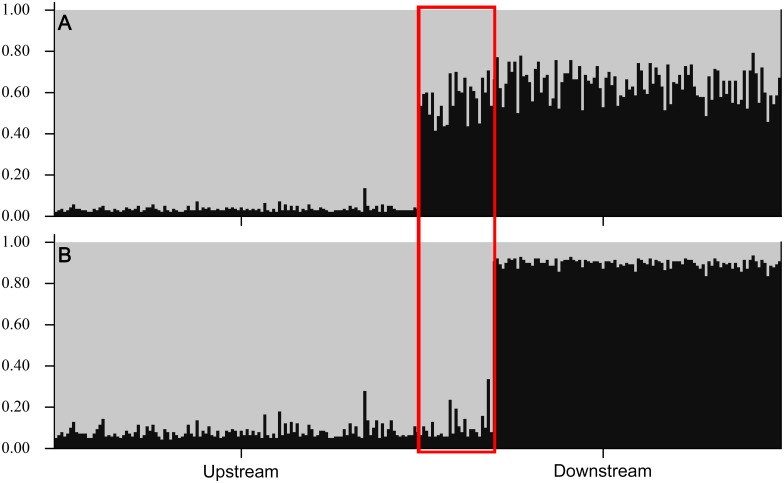
STRUCTURE bar-plots for individual based Bayesian analyses of membership proportions for upstream (n = 124) and downstream (n = 123) sampled juvenile Lake Sturgeon. The two most likely scenarios, (A) k = 2, sampling location as prior (2^nd^ most likely), and (B) k = 2, length-at-age as prior (most likely), are shown. The ordering of individual juveniles is identical for both plots; from left to right, upstream juveniles, fast-growing downstream juveniles (red highlights), and slow-growing downstream juveniles.

When genotype data alone were analyzed using STRUCTURE [114,115], one population (*k* = 1, mean Ln probability of data = -5570) was determined to be the most likely scenario (Fig 4). When sampling location (upstream and downstream) was included as a prior, two populations (*k* = 2, mean Ln probability of data = -5535) were most likely. No upstream immigrants (fast-growing outliers or others) were identified in the downstream population based on membership proportions (Fig 5). Minimum improbability was achieved when two populations (*k* = 2) were considered, and length-at-age (i.e., grouping fast-growing downstream outliers with the upstream fish, see Fig 2) was included as a prior (mean Ln probability of data = -5518). The differences in Ln probability of the data between the two most likely scenarios (#1: length-at-age as prior, *k* = 2 and #2: sampling location as prior, *k* = 2; Fig 4) were highly significant (Steel-Dwass All Pairs, z = 5.7, p <0.0001).

### Population simulations

**Fig 6:**
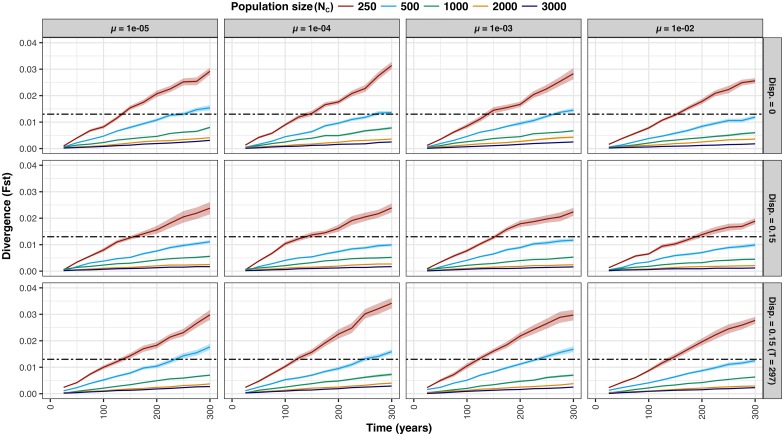
Results of the first 300 years of population divergence simulations (scenarios 1, 2, and 3) conducted in RMETASIM. F_ST_ versus time (years) is plotted based on the 25 year increment mean values derived from 32 simulation replicates conducted for each population size (as shown in the legend), microsatellite mutation rate, and dispersal combination. Shading represents 95% confidence intervals. Horizontal hatched lines reflect the level of F_ST_ observed empirically between upstream and downstream populations after fast-growing outliers were removed from the downstream juvenile group.

**Fig 7:**
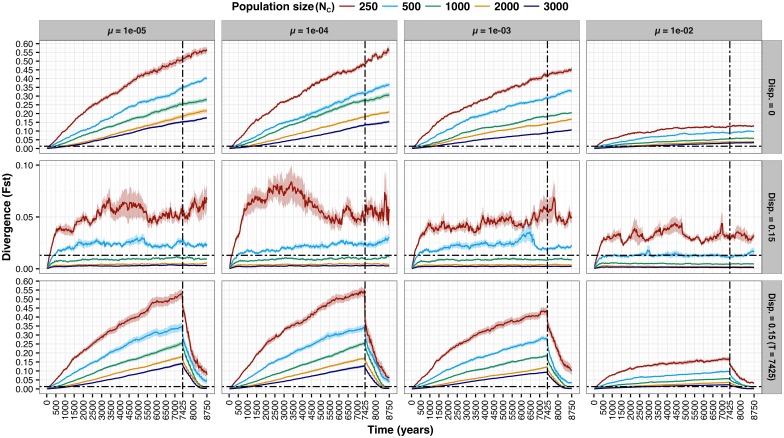
Results of population divergence simulations (scenarios 1, 2, and 3) conducted in RMETASIM. F_ST_ versus time (years) is plotted based on the 25 year increment mean values derived from 32 simulation replicates conducted for each population size (as shown in the legend), microsatellite mutation rate, and dispersal combination. Shading represents 95% confidence intervals. Vertical hatched lines reflect the timing of construction of the Slave Falls GS (~ 7,425 years after glacial recession). Horizontal hatched lines reflect the level of F_ST_ observed empirically between upstream and downstream populations after fast-growing outliers were removed from the downstream juvenile group.

The results of population simulations conducted in RMETASIM [117] are presented in Fig 6 (first 300 years only) and Fig 7 (entire simulation). As expected, rate of F_ST_ divergence decreased monotonically with increasing population size and equilibrium levels of F_ST_ (i.e., asymptotic values) followed the same pattern. There was considerable variation in the rate of F_ST_ divergence and the height of equilibrium plateaus as a function of mutation rate, with 1e^-02^ in particular standing out. Most notably in the context of the historical panmixia null-hypothesis, no parameter combinations yielding F_ST_ > 0.013 (i.e., threshold level determined based on empirical observations with fast-growing outliers excluded) within 100 years were observed (i.e., the approximate timeline since construction of the Slave Falls GS); the fastest attainment of this level occurred in year 125, with N_C_ = 250, dispersal = 0 and mutation rates = 1e^-04^ and 1e^-03^. In Scenario 2, with upstream to downstream dispersal = 0.15 (based on the proportion of fast-growing outliers observed in the downstream population) from the onset of population subdivision, F_ST_ plateaued at values < 0.013 for N_C_ ≥ 1,000. In Scenario 3, the introduction of upstream to downstream dispersal = 0.15 at year 7,425 resulted in a rapid decline of F_ST_ previously accumulated, for all combinations of population size and mutation rate investigated.

## Discussion

In this study, the potential for historical population structure and contemporary inter-reservoir contributions of Lake Sturgeon in the vicinity of a major hydroelectric generating station was assessed using a combination of methods. With regards to contemporary downstream contribution analyses, biological data proved informative. Only small differences in growth rate were observed in the two zones upstream of the Slave Falls GS, despite a ten-fold difference in CPUE, but growth rates of Lake Sturgeon in both upstream zones far exceeded those of the majority of fish located in the riverine section located between Slave Falls and Scot Rapids (Zone DS). It was suspected that all fast-growing outliers were former upstream residents, but because there are areas >15 km downstream of the Slave Falls GS where juvenile Lake Sturgeon exhibit a rapid rate of growth [97], comparable or even higher than what was observed in the Slave Falls Reservoir during the current study, the possibility of upstream redistribution could not be discounted without genetic data.

It has previously been suggested that population genetic results based on juveniles may be biased due to the Allendorf-Phelps effect [128,129]. Essentially, in an age-structured species, if genetic analyses are based on juveniles (not adults), calculated F_ST_ values might be artificially high if only a small number of parents contributed to a cohort that dominates the sample. However, in the current study, upstream and downstream juveniles included in genetic analysis were spread over four and six cohorts, respectively. Three other points also seem relevant: 1) based on census data (Manitoba Hydro unpublished data), it can be reasoned that the quantities of Lake Sturgeon spawning annually in the study area (both below the Pointe du Bois GS and below the Slave Falls GS) are on the order of hundreds; 2) directed studies have found no evidence for sweepstakes reproduction [109] in Lake Sturgeon [107,130]; and, 3) effective population sizes determined for upstream adults (N_e_ = 352 [95% CI: 166–6,683]) and upstream juveniles (N_e_ = 383 [95% CI: 171 - ∞]) in the current study were similarly high.

Most basic comparisons of F_ST_ (i.e., without accounting for putative immigrants) were as hypothesized, with no direct evidence of any Allendorf-Phelps type bias [128,129] due to sampling juveniles; both upstream adults and juveniles were genetically differentiated from downstream juveniles, based on F_ST_ estimates of 0.0108 and 0.0114, respectively. Despite large sample sizes, genetic differentiation of upstream adults to upstream juveniles was not statistically significant and the value calculated was an order of magnitude lower (F_ST_ = 0.00142). No significant genetic differentiation was observed between slow-growing downstream juveniles and the fast-growing outliers, which seems to conflict with the contention that fast-growing outliers were upstream immigrants; if individuals sampled were drawn from the same population, they should be genetically differentiated from slow-growing downstream juveniles. Given the low degree of genetic differentiation observed between Lake Sturgeon upstream and downstream of the Slave Falls GS, this result is likely due to insufficient sample size (i.e., n = 25 fast-growing outliers). In simulations conducted by Kalinowski [131], ~100 individuals per group were required to accurately resolve genetic differentiation when true F_ST_ was 0.01 and 16 highly polymorphic loci were available; the current study only had the benefit of ten loci with an average of 5.9 alleles per locus. However, when fast-growing outliers were excluded from upstream to downstream calculations, differences of mean estimates for key metrics (F_ST_, H_O_, H_E_) between upstream and downstream juvenile groups were more pronounced. Moreover, pairwise relatedness methods as well as Bayesian inference (STRUCTURE) were congruent in indicating inter-reservoir downstream contributions.

Our results highlight the perhaps overlooked utility of combining genetic and biological data (such as length-at-age) when examining structure of fish populations (see reviews [132,133]. Priors are particularly useful when the resolution afforded by available genetic markers is relatively low [114], but it should be stressed that statistical power in these situations will be a function of both the quantity of migrants and overall sample size [134–136], and we suggest that even a low rate of misidentification of putative migrants (e.g., based on biological indicators) are likely to confound interpretation. Further, if the dispersal rate is actually low, large sample sizes may be required to isolate enough migrants to allow for sufficient partitioning of allelic variance; in such cases, methods similar to those followed herein are unlikely to be a replacement for a genetic toolkit that affords the resolution to identify migrants on an individual basis [134,137]. It should be noted that we also explored various other software packages designed to identify population structure and/or isolate migrants (i.e., fast growing outliers) based on genotypes of individuals. GeneClass 2 [138], MavericK 1.0 [139] and discriminant analysis of principal components implemented in adegenet 2.0 [140,141] each employ a different statistical framework, but for the current dataset, all produced results (not shown) that were qualitatively consistent with those of STRUCTURE [114,115].

Our second hypothesis examined the potential for historical population structuring in the study area. At the time of field data collection, only 82 years had passed since construction of the Slave Falls GS began in 1928, which corresponds to a small proportion of the post-glacial period [124–126]. Our simulations showed that, barring very small historical census population sizes (i.e., ≤ 250 individuals per population) and no dispersal between the two populations, an F_ST_ of ≥ 0.013 (i.e., as empirically observed when fast-growing outliers were excluded) would not likely arise within <100 years. Furthermore, based on the results of the current and related studies [90,92], there is evidence of contemporary upstream to downstream contribution, which would theoretically slow the rate of divergence (F_ST_) between populations and also lower the level of equilibrium plateau. It should be noted that considerable uncertainty exists with regards to appropriate parameters for conducting genetic simulations of non-model species with overlapping generations. For example, the understanding of microsatellite mutation rates and repeat restrictions (which we did not incorporate) is incomplete. Also, the nature of regulation for populations at carrying capacity is uncertain and likely varies by species (or perhaps even among habitats inhabited by a given species) from the random approach to thinning that we prescribed in our simulations; in the case of Lake Sturgeon, thinning of juveniles might have different genetic trajectory consequences than would thinning of adults, particularly females, which are generally reputed to attain >75 years of age and be highly fecund late in life [49,54,142,143]. Despite these uncertainties, our simulations offer a first approximation of Lake Sturgeon divergence patterns, and in relation to our primary hypothesis, suggest that population fragmentation <100 years ago would be an unlikely explanation for the levels of upstream and downstream differentiation observed empirically.

Other modelling has also shown that 10–100 generations might be required for fragmentation-induced differentiation to become apparent in species, depending on the size of the fragmented population [20,30]. Lloyd et al. [7] reported that the signal of fragmentation (based on F_ST_) can be present within two generations, but even in species with non-overlapping generations, in populations comprised of ≥500 individuals, the level of differentiation was consistently ≤0.002 and unlikely to be detectable without complete census sampling. Since mean N_e_ estimates in the study area were >350 in the Slave Falls Reservoir (N_C_ estimate: ~2,200 adults as of 2007; Manitoba Hydro unpublished data) and >700 downstream of the Slave Falls GS, and because N_e_ is typically a fraction of N_C_ in fishes [144,145], including Lake Sturgeon [107], it is likely that genetic differentiation observed empirically in the current study would primarily reflect historical processes. As such, it seems unlikely that a previously panmictic population existed in this section of the Winnipeg River immediately prior to construction of a major dam. The mechanisms for minimal (or lack of) historical upstream gene flow are unknown, but the ~6 m plunge found at Old Slave Falls prior to backwatering [86,87], would be the most likely factor given that Lake Sturgeon have poor burst swimming capabilities [146,147].

Perhaps importantly, the degree of upstream to downstream differentiation observed in the current study was lower than has been observed for between-watershed pairings within the Hudson Bay drainage basin, isolated since glacial recession 7500–8500 years B.P. [124–126]. An F_ST_ of 0.1 was observed between Mattagami River (n = 40), Ontario and Rainy River (n = 27), Ontario/Minnesota localities based on the same suite of microsatellites employed herein [75]. Using a similar suite of markers, genetic differentiation between Lake Sturgeon populations was also calculated by McDermid et al. [71]. Considering only pairwise combinations of Attawapiskat River, Burntbush Lake, Rivière Bell, Smoothrock Lake and Saskatchewan River (between-watershed pairings, with sample sizes >30), F_ST_ ranged from 0.04–0.12 [71]. Historical population sizes of Lake Sturgeon in the Winnipeg River and abroad are largely unknown, and population size exerts a strong influence on the rate of inter-population genetic divergence; however, another reasonable explanation for the relatively low level of differentiation (F_ST_ = 0. 013–0.014) between the population upstream and downstream of Slave Falls would be some degree of historical asymmetric gene flow. Previous studies have documented elevated genetic diversity in populations of white spotted-char, *Salvelinus leucomaenis* [148] and river sculpin, *Cottus gobio* [45], located downstream of barriers compared with those isolated upstream. In the current study, observed heterozygosity was greater in juveniles downstream (H_O_ = 0.609, or 0.617 with putative upstream immigrants excluded, as per above) compared with those upstream (H_O_ = 0.556); expected heterozygosity followed a similar pattern.

Some degree of historical upstream to downstream dispersal in the study area seems likely, but the results of Scenario 2 simulations suggest that a 15% rate, ongoing since glacial recession, would not be compatible with the level of differentiation empirically observed if historical population sizes were ≥ 2,000 individuals. Therefore, the quantity of former upstream individuals captured downstream of the Slave Falls GS, in combination with Scenario 3 simulations, raises questions about contemporary persistence of genetic differentiation between upstream and downstream populations. Indeed, the total quantity of downstream juveniles of upstream origin might actually be higher than 15%, as length-at-age data would be unable to identify individuals which passed downstream early in life and subsequently grew at rates characteristic of the downstream sampling location; drifting larvae have been captured immediately upstream of the Slave Falls GS, 9 rkm downstream of the Pointe du Bois spawning site (Manitoba Hydro unpublished data). Given the rapid erosion of accumulated genetic differentiation following the onset of downstream dispersal in Scenario 3 simulations, it seems conceivable that our results may not actually reflect contemporary equilibrium conditions, but rather just a snap-shot of a converging genetic trajectory; a case could be made for population level habitat suitability for Lake Sturgeon having been improved by backwatering from the Slave Falls GS. If so, the Slave Falls Reservoir population may have already recovered from reductions due to historical harvest, and may now actually be approaching a carrying capacity that exceeds what the reach would have supported historically. Therefore, the quantity of upstream to downstream dispersal (relative to *in situ* recruitment in the downstream population) may have changed in the recent past, and could still be in flux; observed and expected heterozygosity were similar for both fast-growing and slow-growing downstream juveniles, and yet the combined downstream juvenile group did not exhibit a heterozygosity deficit, a result which would be consistent with a high-rate of upstream to downstream contribution via large juveniles being a recent phenomenon.

An alternative explanation, also consistent with the lack of heterozygosity deficit in the combined downstream juvenile group, would be that juvenile origin proportions are not indicative of the degree of effective dispersal; differential rates of survival or lifetime fecundity, owing to marked growth rate differences, could be influential. Barth and Anderson [97] found that juveniles between 530 and 879 mm fork length accounted for only 13% of the gillnet catch (n = 2,473) between 2006 and 2008 in the section of river immediately downstream of Slave Falls, perhaps indicative of a population bottleneck. Further, slow-growing downstream residents appear to have plateaued at ~500 mm fork length by age-8, which would actually seem to tilt the balance in favour of fast-growing immigrants from the Slave Falls Reservoir. However, it is unclear if these fish remain fast-growing outliers, or if growth rate declines in their new environment, which can essentially be considered the Slave Falls GS downstream to Scots Rapids due to restricted movement patterns exhibited by the species in Boreal Shield rivers [89,92]. The role of multiple behaviourally isolated juvenile subpopulations residing further downstream in the Seven Sisters Reservoir, some of which exhibit rates of growth exceeding those observed in the Slave Falls Reservoir [89,97], also needs to be considered, because it may be that next-generation contributions of a large quantity of Lake Sturgeon residing downstream of the Slave Falls GS are being superseded by those of a relatively few higher quality individuals, which dispersed further downstream into lacustrine/forebay habitats early in life. Clearly, many questions remain, but our results suggest historical and contemporary processes in this section of the Winnipeg River that are far removed from a simplistic fragmentation-induced divergence scenario, meaning that assessing the true nature of anthropogenic influence (e.g., hydroelectric development, exploitation) likely requires re-thinking several fundamental assumptions in order to direct future analysis appropriately. In the case of Lake Sturgeon, addressing many of the questions pertinent to species recovery will largely be contingent on an improved genetic toolkit.

In summary, genetic approaches in combination with basic biological data (priors) were used to assess historical and contemporary genetic patterns in a small section of a large riverine system developed for hydroelectric power generation. A high rate of inter-reservoir downstream contribution via ongoing entrainment during middle life-stages was observed. Evidence of historical population structure was also revealed. Our results illustrate the importance of establishing a historical baseline, as Lake Sturgeon recovery plans have been focused on the premise of historical within-watershed panmixia and contemporary isolation by dams [61,71,84,85,149–152], a generalization that may not apply to all systems that the species inhabits.

## Supporting information

S1 Dataset Biological and genetic data of Lake Sturgeon captured upstream and downstream of the Slave Falls Generating Station on the Winnipeg River, Manitoba, Canada. (XLSX)

S1 Table Fixed and variable parameters associated with population simulations. (DOCX)

S2 Table Performance comparison of seven pairwise relatedness estimators, based on the ability of the estimators to resolve distributions of known full-sibling, half-sibling, and unrelated Lake Sturgeon, analyzed using a Kruksal-Wallis test, and Steel-Dwass all pairs method for multiple comparisons. High χ^2^ (and low Prob>χ^2^) indicate ability of the various estimators to resolve overall differences in relatedness distributions. Probabilities associated with multiple comparison tests (e.g., full-sib versus half-sib) are also shown; however, all values were similarly low and therefore the metric largely uninformative. Estimators are ranked from left to right.
(DOCX)

S3 Table Comparison of mean relatedness of Upstream (US) and Downstream (DS) juveniles based on seven relatedness estimators. Data is summarized by mean relatedness (r) and Standard Deviation of relatedness (SD). Groups that share the same score in the significance (Sig) columns did not have statistically different distributions, as determined by a Wilcoxon Rank-Sum test. Estimators are listed from left to right in order of performance ranking (S2 Table).
(DOCX)

S4 Table Comparison of mean relatedness of Upstream (US), fast-growing Downstream (DS fast) and slow-growing Downstream (DS slow) fish with the upstream juvenile group, based on seven relatedness estimators and analyzed using Kruksal-Wallis. Data is summarized by mean relatedness (r) and standard deviation of relatedness (SD). Groups that share the same score in the significance (Sig) columns did not have statistically different distributions, as determined by Steel-Dwass All Pairs method. Estimators are listed from left to right in order of performance ranking (S2 Table).
(DOCX)
