# Supplementary material for: Rethinking the influence of hydroelectric development on gene flow in a long-lived fish, the Lake Sturgeon Acipenser fulvescens
Source: PLoS One. 2017 Mar 22;12(3):e0174269. doi: 10.1371/journal.pone.0174269 (PMC5362236; doi:10.1371/journal.pone.0174269)
Supplement: S1 Table — (DOCX) [file pone.0174269.s002.docx]

**S1 Table. Fixed and variable parameters associated with population simulations.**

| Parameter | Fixed values | Varied values |
| --- | --- | --- |
| Population size (carrying capacity) | - | 250, 500, 1000, 2000, 3000 |
| Population sex ratio | 1:1 | - |
| Initial allelic frequency distribution | Empirically observed |  |
| Mutation scheme | Single-step | - |
| Mutation rate (µ) | - | 1e^-05^, 1e^-04^, 1e^-03^, 1e^-02^ |
| Dispersal rate | - | 0, 0.15@year 0, 0.15@year 7425 |
| Annual survival rate | age 1-24: 0.986, age 25-100: 0.95, age 100: 0 | - |
| Female reproduction | 0.3 @ 4 year breeding interval | - |
| Male reproduction | 0.05263158 @ 2 year breeding interval | - |
