# Supplementary material for: Rethinking the influence of hydroelectric development on gene flow in a long-lived fish, the Lake Sturgeon Acipenser fulvescens
Source: PLoS One. 2017 Mar 22;12(3):e0174269. doi: 10.1371/journal.pone.0174269 (PMC5362236; doi:10.1371/journal.pone.0174269)
Supplement: S2 Table — High χ2 (and low Prob>χ2) indicate ability of the various estimators to resolve overall differences in relatedness distributions. Probabilities associated with multiple comparison tests (e.g., full-sib versus half-sib) are also shown; however, all values were similarly low and therefore the metric largely uninformative. Estimators are ranked from left to right. (DOCX) [file pone.0174269.s003.docx]

**S2 Table. Performance comparison of seven pairwise relatedness estimators, based on the ability of the estimators to resolve distributions of known full-sibling, half-sibling, and unrelated Lake Sturgeon, analyzed using a Kruksal-Wallis test, and Steel-Dwass all pairs method for multiple comparisons.** High χ^2^ (and low Prob>χ^2^) indicate ability of the various estimators to resolve overall differences in relatedness distributions. Probabilities associated with multiple comparison tests (e.g., full-sib versus half-sib) are also shown; however, all values were similarly low and therefore the metric largely uninformative. Estimators are ranked from left to right.

| Statistic | Lynch and Ritland | TrioML | Milligan | Lynch | Queller | Wang | Ritland |
| --- | --- | --- | --- | --- | --- | --- | --- |
| χ^2^ | 428.2 | 418.1 | 414.7 | 370.4 | 354.3 | 353.6 | 326.4 |
| Prob>χ^2^ | <0.0001 | <0.0001 | <0.0001 | <0.0001 | <0.0001 | <0.0001 | <0.0001 |
| Full-sib vs. half-sib p-value | 0.0004 | <0.0001 | <0.0001 | <0.0001 | <0.0001 | <0.0001 | <0.0001 |
| Full-sib vs. unrelated p-value | <0.0001 | <0.0001 | <0.0001 | <0.0001 | <0.0001 | <0.0001 | <0.0001 |
| Half-sib vs. unrelated p-value | <0.0001 | <0.0001 | <0.0001 | <0.0001 | <0.0001 | <0.0001 | <0.0001 |
