# Supplementary material for: Rethinking the influence of hydroelectric development on gene flow in a long-lived fish, the Lake Sturgeon Acipenser fulvescens
Source: PLoS One. 2017 Mar 22;12(3):e0174269. doi: 10.1371/journal.pone.0174269 (PMC5362236; doi:10.1371/journal.pone.0174269)
Supplement: S3 Table — Groups that share the same score in the significance (Sig) columns did not have statistically different distributions, as determined by a Wilcoxon Rank-Sum test. Estimators are listed from left to right in order of performance ranking (S2 Table). (DOCX) [file pone.0174269.s004.docx]

**S3 Table. Comparison of mean relatedness of upstream (US) and downstream (DS) juveniles based on seven relatedness estimators. Data is summarized by mean relatedness (r) and standard deviation of relatedness (SD).** Groups that share the same score in the significance (Sig) columns did not have statistically different distributions, as determined by a Wilcoxon Rank-Sum test. Estimators are listed from left to right in order of performance ranking (S2 Table).

| Location | Lynch & Ritland | | | TrioML | | | Milligan | | | Lynch | | | Queller | | | Wang | | | Ritland | | |
| --- | --- | --- | --- | --- | --- | --- | --- | --- | --- | --- | --- | --- | --- | --- | --- | --- | --- | --- | --- | --- | --- |
|  | r | SD | Sig | r | SD | Sig | r | SD | Sig | r | SD | Sig | r | SD | Sig | r | SD | Sig | r | SD | Sig |
| US | 0.011 | 0.025 | A | 0.087 | 0.025 | A | 0.111 | 0.027 | A | 0.010 | 0.128 | A | 0.026 | 0.075 | A | -0.007 | 0.118 | - | 0.01 | 0.029 | - |
| DS | 0.005 | 0.02 | B | 0.075 | 0.018 | B | 0.100 | 0.022 | B | -0.024 | 0.109 | B | -0.013 | 0.065 | B | -0.011 | 0.104 | - | 0.01 | 0.033 | - |
