# Supplementary material for: Rethinking the influence of hydroelectric development on gene flow in a long-lived fish, the Lake Sturgeon Acipenser fulvescens
Source: PLoS One. 2017 Mar 22;12(3):e0174269. doi: 10.1371/journal.pone.0174269 (PMC5362236; doi:10.1371/journal.pone.0174269)
Supplement: S4 Table — Data is summarized by mean relatedness (r) and standard deviation of relatedness (SD). Groups that share the same score in the significance (Sig) columns did not have statistically different distributions, as determined by Steel-Dwass All Pairs method. Estimators are listed from left to right in order of performance ranking (S2 Table). (DOCX) [file pone.0174269.s005.docx]

**S4 Table. Comparison of mean relatedness of upstream (US), fast-growing downstream (DS fast) and slow-growing downstream (DS slow) fish with the upstream juvenile group, based on seven relatedness estimators and analyzed using Kruksal-Wallis.** Data is summarized by mean relatedness (r) and standard deviation of relatedness (SD). Groups that share the same score in the significance (Sig) columns did not have statistically different distributions, as determined by Steel-Dwass All Pairs method. Estimators are listed from left to right in order of performance ranking (S2 Table).

| Location | Lynch & Ritland | | | TrioML | | | Milligan | | | Lynch | | | Queller | | | Wang | | | Ritland | | |
| --- | --- | --- | --- | --- | --- | --- | --- | --- | --- | --- | --- | --- | --- | --- | --- | --- | --- | --- | --- | --- | --- |
|  | r | SD | Sig | r | SD | Sig | r | SD | Sig | r | SD | Sig | r | SD | Sig | r | SD | Sig | r | SD | Sig |
| US | 0.011 | 0.025 | A | 0.087 | 0.025 | A | 0.111 | 0.027 | A | 0.010 | 0.128 | A | 0.026 | 0.075 | A | -0.007 | 0.118 | A,B | 0.010 | 0.029 | A |
| DS fast | 0.003 | 0.026 | A | 0.088 | 0.025 | A | 0.110 | 0.027 | A | 0.035 | 0.108 | A | 0.034 | 0.076 | A | 0.022 | 0.101 | A | -0.003 | 0.031 | A |
| DS slow | -0.021 | 0.025 | B | 0.064 | 0.023 | B | 0.084 | 0.027 | B | -0.044 | 0.126 | B | -0.025 | 0.076 | B | -0.041 | 0.115 | B | -0.023 | 0.033 | B |
